# SparRec: An effective matrix completion framework of missing data imputation for GWAS

**DOI:** 10.1038/srep35534

**Published:** 2016-10-20

**Authors:** Bo Jiang, Shiqian Ma, Jason Causey, Linbo Qiao, Matthew Price Hardin, Ian Bitts, Daniel Johnson, Shuzhong Zhang, Xiuzhen Huang

**Affiliations:** 1 Research Center for Management Science and Data Analytics, School of Information Management and Engineering, Shanghai University of Finance and Economics, Shanghai 200433, China; 2 Department of Systems Engineering and Engineering Management, The Chinese University of Hong Kong, Shatin, N.T., Hong Kong; 3 Department of Computer Science, Arkansas State University, Jonesboro, Arkansas 72467, United States of America; 4 The UALR/UAMS Joint Graduate Program in Bioinformatics, Little Rock, Arkansas 72204, United States of America; 5 College of Computer, National University of Defense Technology, Changsha 410073, China; 6 Molecular Biosciences Program, Arkansas State University, Jonesboro, Arkansas 72467, United States of America; 7 The University of Tennessee Health Science Center (UTHSC) rBIO Core Lab, Cancer Research Building Rm258, 19 S Manassas St. Memphis, TN 38163, United States of America; 8 Department of Industrial and Systems Engineering, University of Minnesota, Minneapolis, Minnesota 55455, United States of America

## Abstract

Genome-wide association studies present computational challenges for missing data imputation, while the advances of genotype technologies are generating datasets of large sample sizes with sample sets genotyped on multiple SNP chips. We present a new framework SparRec (Sparse Recovery) for imputation, with the following properties: (1) The optimization models of SparRec, based on low-rank and low number of co-clusters of matrices, are different from current statistics methods. While our low-rank matrix completion (LRMC) model is similar to Mendel-Impute, our matrix co-clustering factorization (MCCF) model is completely new. (2) SparRec, as other matrix completion methods, is flexible to be applied to missing data imputation for large meta-analysis with different cohorts genotyped on different sets of SNPs, even when there is no reference panel. This kind of meta-analysis is very challenging for current statistics based methods. (3) SparRec has consistent performance and achieves high recovery accuracy even when the missing data rate is as high as 90%. Compared with Mendel-Impute, our low-rank based method achieves similar accuracy and efficiency, while the co-clustering based method has advantages in running time. The testing results show that SparRec has significant advantages and competitive performance over other state-of-the-art existing statistics methods including Beagle and fastPhase.

Genome-wide association studies (GWAS) are promising to contribute to uncovering the genetic variations for many complex human diseases, with the many initiatives including the International HapMap Project and the 1000 Genomes Project. Genotype imputation represents the computational challenge of predicting the genotypes at the SNPs that are not directly genotyped in the study sample. Genotype imputation could increase the power of the GWAS study through fine-mapping to increase the chance of a causal SNP being identified, meta-analysis when combining different cohorts using different genotyping chips, and imputation of untyped variations which are not typed in the reference panel or the study sample^1^. Genotype imputation methods have been widely used to boost genome-wide association studies across the genome or at a focused region.

With the advances of genotyping technologies, large amounts of genetic datasets are being continuously and rapidly generated around the world. As is pointed in ref. 2, for “next-generation” association data studies in the near future, we will be handling datasets with (1) larger sample sizes, (2) unphased and incomplete genotypes, and (3) multiple reference panels and more diverse study datasets from different platforms with different sets of SNPs. This brings great computational challenges to the genotype imputation problem, which calls for not only the improvement of the current approaches, but also new mathematical models, which are supposed to exploit the structure of the large datasets and model the imputation problem in ways different from traditional approaches.

Many current imputation methods are based on statistics models, such as the Hidden Markov Model (HMM) and the Expectation-Maximization (EM) algorithm^1^,^3^. The way they work is to use haplotype patterns in reference panels to predict untyped genotypes in study panels. They depend on the availability of the reference panels, thus their performances are in a way limited by the quality of the chosen reference panels. Due to the nature of the models, current statistics methods are not flexible in handling datasets with multiple reference panels and diverse study panels for imputation tasks with various studies. Current statistics methods often require a cumbersome process to prepare the formatted input files, as well as a process for interpreting the output results from the computational methods, which makes the approaches less amenable to biological and biomedical researchers imputing their study samples.

Chi *et al*.^4^ developed a low-rank matrix completion based method Mendel-Impute for GWAS imputation. The HMM or EM based statistics methods and the low-rank matrix completion based method Mendel-Impute all rely on the identification of shared haplotypes (or the low-rank structure) in local blocks arising from linkage disequilibrium (LD). For the computational models proposed in this paper, our idea originated from the observation that there are often some sort of sparsity structures, such as low-rank and/or low numbers of co-clusters, in large genomic data matrices. Though similar sparsity structure has been used in imaging processing in the context of compressive sensing, only until recently we applied it in analyzing cancer patient gene expression profiling data matrices^5^. In this paper we propose to use the sparsity structure to meet the challenge of missing data imputation for GWAS.

In this paper we present a new framework, SparRec (Sparse Recovery), for imputing missing genetic data in genome-wide association studies. The models of SparRec are designed based on the sparse properties of low-rank and low numbers of co-clusters of the large, noisy, genetic datasets of matrices with missing data. We would like to point out that the low-rank matrix completion (LRMC) model is similar to Mendel-Impute, but the matrix co-clustering factorization (MCCF) model is completely new. We will illustrate how our approach is able to effectively find patterns for imputation within study data, both with and without reference panels, and even with data missing rate as high as 90%. We will compare the performance of our approach with several other mainstream approaches for genotype imputation, including statistics methods fastPHASE^6^ and Beagle^7^, and the low-rank matrix completion based method Mendel-Impute^4^. SparRec is easy to use for metadata analysis, and it requires very simple, easy-to-process input file format and easy-to-interpret output result files. It has better or comparable performance compared to current state-of-the-art methods, especially for handling large sample size data with very different sets of SNPs and no reference panels.

## Methods

### The problem of genotype imputation

Current approaches for haplotype inference and missing data imputation usually process the genotype data, and each sample is phased and the haplotypes are modeled as mosaic of those haplotypes of the reference panel^1^,^3^. Our approach could conduct imputation for both haplotype and genotype data matrices from different cohorts using different genotyping chips.

**Figure 1:**
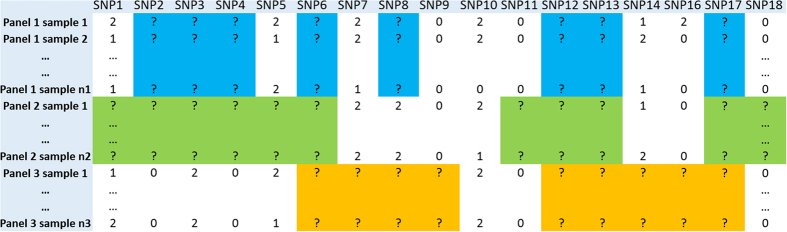
The genotype data matrix with three reference or study panels with missing data at color-highlighted untyped SNPs, generated by three different cohorts using different genotyping chips.

**Figure 2:**
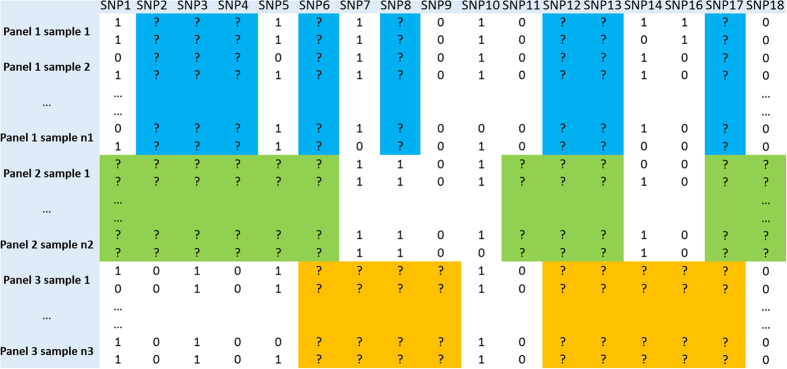
The haplotype data matrix with color-highlighted missing data entries.

Given the matrices of the genotype data with untyped SNPs or the phased haplotype data with missing values, the imputation problem is to impute the missing data entries of the data matrices. The reader is referred to the illustrative formats of the data matrices in Figs 1 and 2 for the diploid genotype data and the corresponding phased haplotype data. For the data matrices, each row corresponds to one individual sample and each column corresponds to one SNP. In the genotype data matrices, 0, 1, and 2, which correspond to the number of minor alleles that an individual carries, are used to represent the possible genotypes such as, BB, Bb, bb, with B and b being A, T, C or G. In the haplotype data matrices, 0 and 1 are used to represent the major allele and the minor allele.

We present a natural and flexible modelling framework, which utilizes information across multiple reference panels and study panels, and which achieves high recovery accuracy even when the data matrices have high percentages of missing entries. Our approach combines the multiple chosen reference panels and the different study panels together as a large whole data matrix with missing entries (refer to Figs 1 and 2). The idea of our approach is based on the following observation: although the large genotype data matrix, which usually has missing entries and which may be contaminated by noises and errors from experimental samples and sequencing technologies, appear to be very complex, the underlying structure of the data matrix contains essential “sparse” information. By “sparse”, we mean the data matrix has the mathematical property of low-rank or low number of co-clusters. We test the sparse property using large genotype data matrices and the testing results show the matrices are usually low-ranked. In the following we present the technical details of our approach, and test the effects of different matrix numerical representations to the ranks of data matrices.

### Imputation Based on Low-Rank Matrix Completion

The low-rank matrix completion (LRMC) model aims to fill in missing data values of a matrix based on the priori information that the matrix under consideration is of low rank. The low-rank matrix completion model can be formulated as the following optimization problem:


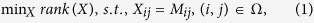


where *rank*(*X*) denotes the rank of matrix *X*, and Ω denotes the index set of the known entries of *M*. That is, we are given a set of known entries of *M*, and we want to fill in the missing entries such that the completed matrix is of low rank. In the genotype missing data imputation problem, each row of the matrix *M* represents a patient sample, and each column of the matrix *M* corresponds to a SNP. That is, *M*_*ij*_ represents the *j*-th allele of the *i*-th patient sample. It is usually believed that patients can be classified into different categories and patients in the same category should have similar genetic patterns. Therefore, we believe that the matrix *M* is low-rank, or at least numerically low-rank.

The LRMC model has been widely used in online recommendation, collaborative filtering, computer vision and so on. Recently, it has been shown in refs 8, 9, 10, that under certain randomness hypothesis, the model (1) is equivalent to the following convex optimization problem with high probability:


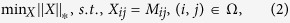


where ||*X*||_*_ is called the nuclear norm of matrix *X* and is defined as the sum of singular values of *X*. The nuclear norm minimization problem (NNM) is numerically easier to solve than the LRMC model because it is a convex problem. Many efficient numerical algorithms have been suggested to solve the NNM model, for example^11^,^12^,^13^, to name just a few. In this paper, we use the fixed point continuation method (FPCA) proposed in ref. 11 to solve the model (2).

A closely related work to (LRMC) is the Mendel-Impute method introduced in ref. 4. The Mendel-Impute method implements Nesterov’s accelerated proximal gradient method (APG) to solve (2), while FPCA proposed in ref. 11 can be seen as the ordinary version of proximal gradient method for solving (2). Theoretically, APG is faster than FPCA for solving LRMC, because the former attains an 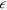-optimal solution in 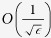 iterations, while the latter one attains an 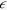-optimal solution in 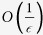 iterations. Mendel-Impute also implements two important techniques to further accelerate the speed of APG: the sliding window scheme to better balance the trade-offs between accuracy and running time, and the line search technique to find an appropriate step size for the proximal gradient step. From our experiments, we found that the sliding window scheme is quite helpful for missing data imputation. Thus, we incorporated the sliding window scheme to LRMC, denoted as LRMC-s. We observed from our numerical tests that the performance of LRMC-s is comparable to Mendel-Impute.

### Imputation Based on Matrix Co-clustering Factorization

We now propose a new approach for imputation that is based on matrix co-clustering factorization (MCCF). Ma *et al*. in ref. 5 developed a co-clustering model for two-dimensional and higher-dimensional matrix co-clustering, which is based on a tensor optimization model and an optimization method termed Maximum Block Improvement (MBI)^14^,^15^. Inspired by the idea of matrix co-clustering for imputation, we develop a basic model as follows.


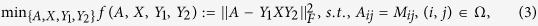


where 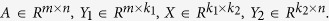


In (3), the Frobenius norm of a matrix X is defined as 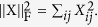 Our imputation approach, based on the matrix co-clustering factorization, aims to complete matrix *M* by using a low-rank matrix factorization model. In our framework, *A* is the data matrix with missing entries; *Y*_1_ and *Y*_2_ are the artificial row assignment matrix and the artificial column assignment matrix, respectively, and *X* is the artificial central-point matrix. Note that A is also an unknown decision variable in (3), because only a subset of its entires is known. Moreover, note that (3) requires the input of *k*_1_ and *k*_2_, which are closely related to the rank of the matrix to be completed. Therefore, in practice, if we have a good estimation to the rank of the matrix, then (3) is a better model to use than (2), because it also provides us the clustering information of individual samples and SNPs.

**Figure 3:**
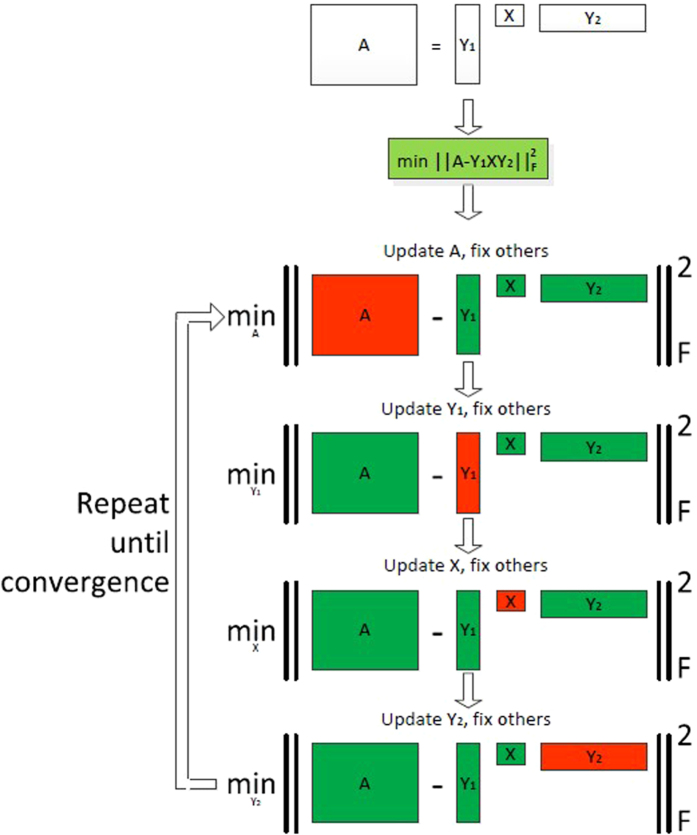
The flow chart of the imputation algorithm based on the idea of matrix co-clustering factorization (MCCF). A is the data matrix with missing entries; Y_1 and Y_2 are the artificial row assignment matrix and the artificial column assignment matrix, respectively, and X is the artificial central-point matrix.

Although the MCCF model is non-convex, it has some natural block-structure that can be utilized to adopt an efficient solution method. We propose to solve the model (3) using a block coordinate update (BCU) procedure. There are four block variables in the model (3), namely *A, X, Y*_1_ and *Y*_2_. The basic idea of BCU is, at each iteration, to minimize the function *f* with respect to one block variable while the other three blocks are fixed at the current known values. This idea is effective because we observed that minimizing *f* for only one block variable among *A, X, Y*_1_ and *Y*_2_ is always relatively easy. A naive implementation of the BCU idea is to minimize *f* in the order of *A* → *Y*_1_ → *X* → *Y*_2_, and in each step only one block variable is updated with the other three blocks being fixed. The flow chart of this BCU algorithm is shown in Fig. 3. In our MCCF model, the matrix *X* actually plays a more important role than the other three blocks. As a result, it is beneficial if we can update the *X* block more frequently than the other three blocks. Therefore, we implemented the following four different algorithms based on the BCU idea.

1. “BCU-1”: BCU with Block Loop in the following order (*A* − *X*) → (*Y*_1_ − *X*) → (*Y*_2_ − *X*). That is, we take *A* and *X* together as one (bigger) block. Similarly *Y*_1_ and *X* is one block and *Y*_2_ and *X* is one block. For these three blocks, we use BCU to update them one by one. In each block, for example, (*A* − *X*), since we need to minimize *f* with respect to *A* and *X* simultaneously, we use the alternating minimization procedure that minimizes *f* with respect to *A* and *X* alternatingly, until the function value ceases to change. The other two blocks (*Y*_1_ − *X*) and (*Y*_2_ − *X*) are dealt with in the same way.
2. “BCU-2”: BCU with the following order of block variables: *A* → *X* → *Y*_1_ → *X* → *Y*_2_. That is, we update *X* twice in each sweep of the block variables.
3. “BCU-3”: BCU with the following order of block variables: *A* → *X* → *Y*_1_ → *X* → *Y*_2_ → *X*. That is, we update *X* three times in each sweep of the block variables. This variant of BCU has been considered by Xu^16^ for tensor decomposition problems.
4. “MBI-BL”: This is a variant of the MBI algorithm proposed by Chen *et al*.^15^. MBI-BL applies MBI algorithm in ref. 15 to minimize *f* with four blocks variables: 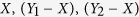 and (*A* − *X*). In each block, for example, (*A* − *X*), we use alternating block minimization scheme to minimize *f* with respect to *A* and *X* alternatingly, until the function value ceases to change. After having attempted all four block variables, we update the block variable with maximum improvement.

All the algorithms are terminated when the objective value in the (*k* + 1)-th iteration does not decrease significantly from that in the *k*-th iteration. We give the detailed description of BCU-1, BCU-2, BCU-3 and MBI-BL as Algorithms 1, 2, 3 and 4, respectively (Refer to the steps of each of the four algorithms as follows).

**Algorithm 1 (BCU-1)**

Given initial iterates 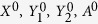 and initial values *v*_0_ = 0, *v*_1_ = 1:

For *k* = 0, 1, …, run the following until 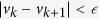


1) (Update *A* and *X*). For 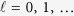 run the following updates I and II for *A* and *X* until the objective value does not change much; *X*^*k*,0^ = *X*^*k*^

I. 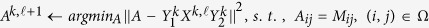


II. 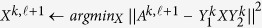


2) Set 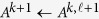, 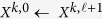


3) (Update *Y*_1_ and *X*). For 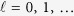 run the following updates I and II for *Y*_1_ and *X* until the objective value does not change much:

I. 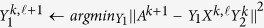


II. 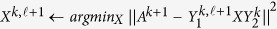


4) Set 
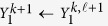
, 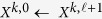


5) (Update *Y*_2_ and *X*). For 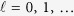 run the following updates I and II for *Y*_2_ and *X* until the objective value does not change much:

I. 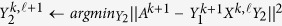


II. 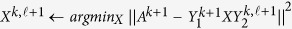


6) Set 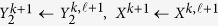


7) Compute 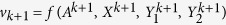


**Algorithm 2 (BCU-2)**

Given initial iterates 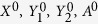 and initial values *v*_0_ = 0, *v*_1_ = 1:

For *k* = 0, 1, …, run the following until 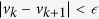

1. Update *A*: 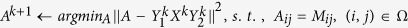
2. Update *X*: 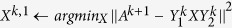
3. Update *Y*_1_: 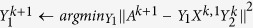
4. Update *X*: 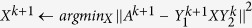
5. Update *Y*_2_: 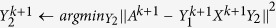
6. Compute 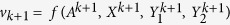

**Algorithm 3 (BCU-3)**

Given initial iterates 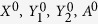 and initial values *v*_0_ = 0, *v*_1_ = 1:

For *k* = 0, 1, …, run the following until 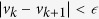

1. Update *A*: 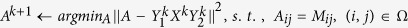
2. Update *X*: 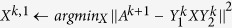
3. Update *Y*_1_: 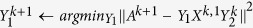
4. Update *X*: 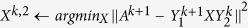
5. Update *Y*_2_: 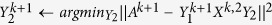
6. Update *X*: 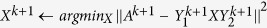
7. Compute 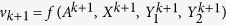

**Algorithm 4 (MBI-BL)**

Given initial iterates 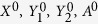, and initial values *v*_0_ = 0, *v*_1_ = 1.

For *k* = 0, 1,…, run the following until 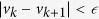

1. Block Improvement:
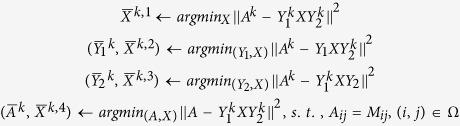
2. Compute the corresponding objective values:
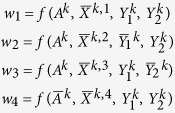
3. Maximum Improvement: Compare *w*_1_, *w*_2_, *w*_3_, *w*_4_, pick up the smallest value to update the corresponding block variables:
  - If *w*_1_ is the smallest, then 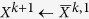, *v*_*k*+1_ ← *w*_1_
  - If *w*_2_ is the smallest, then 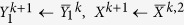, *v*_*k*+1_ ← *w*_2_
  - If *w*_3_ is the smallest, then 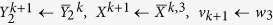  - If *w*_4_ is the smallest, then 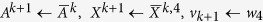

### The effects of different matrix numerical representations to the matrix ranks

**Figure 4:**
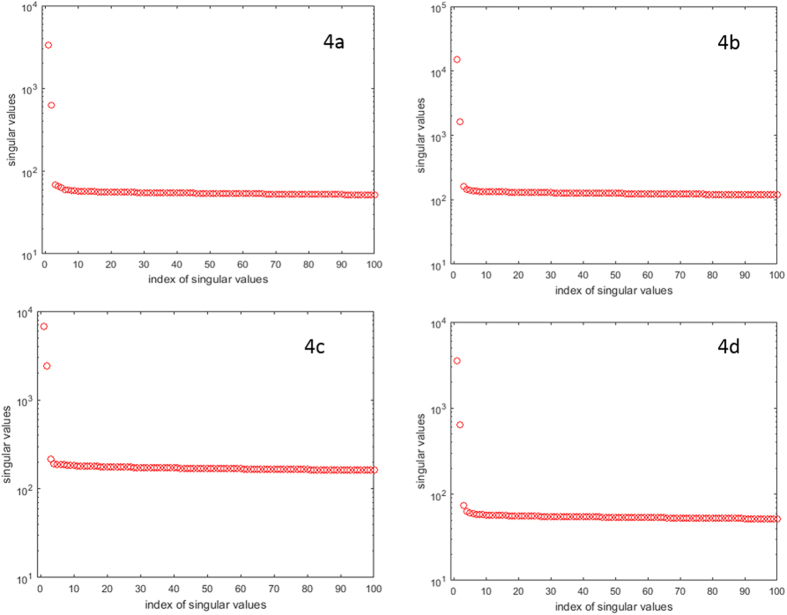
The effects of different numerical representations to the matrix ranks, with the matrix of size (10,000 × 3,000). **Part a** (top left): the distribution of the 100 leading singular values of the data matrix obtained by assigning A = 0, T = 1, C = 0, G = 1 in data-00-ATCG. **Part b** (top right): the distribution of the 100 leading singular values of the data matrix obtained by assigning A = 1, T = 2, C = 3, G = 4 in data-00-ATCG. **Part c** (bottom left): the distribution of the 100 leading singular values of the data matrix obtained by assigning A = −2, T = −1, C = 1, G = 2 in data-15-ATCG. **Part d** (bottom right): the distribution of the 100 leading singular values of the data matrix obtained by assigning A = 1, T = 0, C = 1, G = 0 in data-15-ATCG.

**Figure 5:**
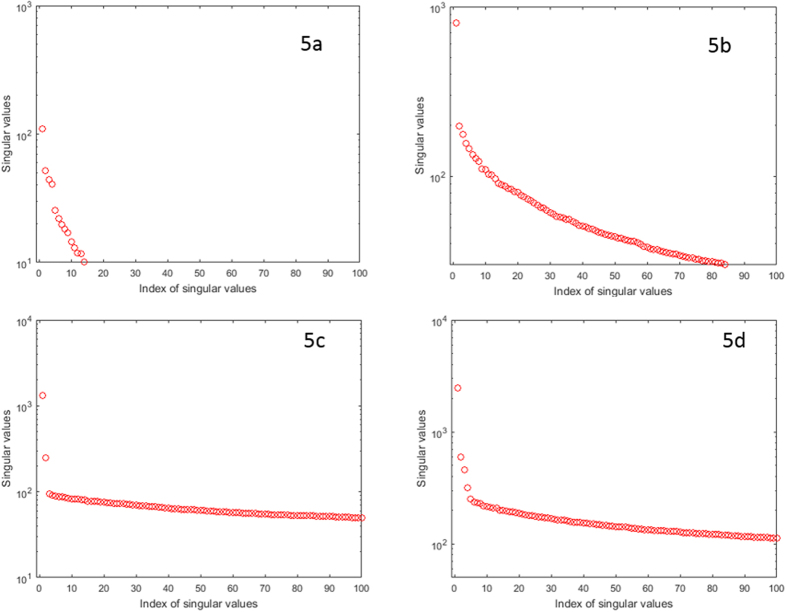
The leading singular values of four datasets. **Part a**: Chr22, **Part b**: Chr22_3841, **Part c**: HapMap3, and **Part d**: 1KG_Chr22. From these figures we can see that these datasets are approximately of low rank.

Here we show that the way we represented the entries of the matrix with different numbers seems to generate approximate low-rank matrices in practice. These observations justified that our (LRMC-s) and (MCCF) models indeed capture the underlying structures of the data. We used two data sets referred to as “data-00-ATCG” and “data-15-ATCG” in our experiment: Both data sets are of size (10,000 × 3,000), with each data set including 10,000 alleles and 1,500 sample diplotypes from the British Birth Cohort. We computed the singular values of the data matrix obtained by assigning A = 0, T = 1, C = 0, G = 1 in data-00-ATCG, and the top ten leading singular values are 1e + 3*[3.3236, 0.6248, 0.0687, 0.0660, 0.0635, 0.0599, 0.0592, 0.0582, 0.0578, 0.0574]. We can see that the largest singular value is about 48 times bigger than the third largest singular value, the second largest singular value is about 9 times bigger than the third largest singular value. However, starting from the third singular value, there is not much difference among the rest singular values. As a result, we can conclude that this data matrix is approximately a rank-2 matrix. To help the readers better visualize this observation, we plot the 100 leading singular values of this data matrix in Fig. 4a, from which we can clearly see that the first two singular values are significantly larger than other singular values and the matrix can be viewed as an approximately rank-2 matrix. Similarly, Fig. 4b shows the 100 leading singular values of the data matrix obtained by assigning A = 1, T = 2, C = 3, G = 4 in data-00-ATCG, whose 10 leading singular values are 1e + 4*[1.5100, 0.1621, 0.0159, 0.0143, 0.0142, 0.0139, 0.0136, 0.0135, 0.0135, 0.0133], which again indicates that the resulting matrix can be regarded as an approximate rank-2 matrix. Figure 4c shows the 100 leading singular values of the matrix obtained by assigning A = −2, T = −1, C = 1, G = 2 in data-15-ATCG, whose 10 leading singular values are 1e + 3*[6.7762, 2.3994, 0.2154, 0.1906, 0.1879, 0.1866, 0.1855, 0.1842, 0.1830, 0.1819], which again indicates that the resulting matrix is approximately rank-2. Figure 4d shows the 100 leading singular values of the matrix obtained by assigning A = 1, T = 0, C = 1, G = 0 in data-15-ATCG, whose leading 10 singular values are 1e + 3*[3.5305, 0.6383, 0.0746, 0.0630, 0.0601, 0.0596, 0.0585, 0.0580, 0.0578, 0.0575], which again indicates that the resulting matrix is approximately rank-2. Moreover, we also computed the leading singular values of datasets Chr22, Chr22_3841, HapMap3 and 1KG_chr22 are the results are shown in Fig. 5. From Fig. 5 we can see that these datasets are approximately low-rank as well. All these observations suggest that the resulting matrices are usually low-rank, and thus can be completed by solving our models (LRMC-s) and (MCCF) using the proposed methods.

For LRMC-s and MCCF, they are two different models for the same target: matrix completion. Usually if we have a good estimation of the rank of the matrix, then we can decide the size of matrices Y_1_, X, Y_2_, and thus MCCF is preferred because it also provides the clustering information of individual samples and SNPs. Otherwise, we should use LRMC-s, which does not need the rank information.

### Data availability

The framework of SparRec (Sparse Recovery) is implemented in MATLAB and the source code is available from: https://sourceforge.net/projects/sparrec/files/?source=navbar Or, http://bioinformatics. astate.edu/code2/ and also available on GitHub at: https://github.com/astate-bioinformatics/SparRec.

## Testing Results

In this section, we shall test the recovering capabilities of all of our proposed methods and the three currently mainstream imputation methods (Beagle, fastPHASE and Mendel-Impute) for several real datasets with or without reference information. In Tables 1, 2, 3, 4, 5, 6, we report the error rates of fastPHASE, Beagle, Mendel-Impute, LRMC-s, and four MCCF algorithms: BCU-1, BCU-2, BCU-3 and MBI-BL, where we chose *k*_1_ = 100 and *k*_2_ is the one that gives the best error rate from the list {2, 3, 5, 10, 20}, and in most cases *k*_2_ = 20. Moreover, in Tables 7 and 8, we report the comparison of these algorithms for various datasets with 1% masked genotypes, which are commonly encountered in GWAS study. Overall, we shall see that Mendel-Impute and LRMC-s are quite comparable, and outperform other methods. In Tables 9, 10, 11, 12, 13, we test the robustness of our four MCCF algorithms with different combinations of *k*_1_ and *k*_2_, and our results show that the MCCF algorithms are not sensitive to *k*_1_, and larger *k*_2_ usually gives better error rates.

### Comparison of our LRMC-s and MCCF algorithms with Mendel-Impute, Beagle and fastPHASE

To justify the performance of our proposed methods, we compare the recovery accuracy of our algorithms with that of some popular statistics imputation method such as Beagle^7^ (version 3.3.2), fastPHASE^6^ (version 1.4.0). Both of those methods belong to haplotype-inference methods. In particular, fastPHASE is based on a haplotype-clustering model with a fixed number of clusters, while Beagle describes the correlation between markers as a localized haplotype-cluster model, which can in turn be viewed as a hidden Markov model (HMM). The merit of HMM is that it can be used to sample or find the most likely haplotype pair for each individual. Different from other approaches (such as IMPUTE v2^2^), Beagle and fastPHASE still work even in the absence of reference panels, making them an ideal candidate for comparison. We also compare our approaches with another low-rank matrix completion based method Mendel-Impute^4^ in the experiment.

#### Criteria for Evaluation

For each data set, we first masked 5%, 10%, 25%, 50%, 60%, 70%, 80% and 90% of the genotypes respectively. Then we run fastPHASE, Beagle (default setting with R = 4) and our methods proposed above together with some rounding procedure if necessary to recover the missing data. The raw output of our methods consists of numbers in the range 0–2. These are converted into genotypes by rounding and an estimate of uncertainty could be obtained by means of a similar post-processing step to that used in ref. 4.

The so-called allelic-imputation error rate is referred to as the proportion of missing alleles that are incorrectly imputed. It has been widely adopted in the literature to measure the capability of various imputation approaches. In this paper, we calculated this error rate of the tested methods and summarize the results in the following tables.

In Tables 1, 2, 3, 4, 5, 6, we reported the comparison results of Mendel-Impute, LRMC-s and MCCF on various datasets. We shall see that the performance of Mendel-Impute and LRMC-s is quite comparable.

#### Comparison without Reference Information

The first three datasets used for comparison are tested without using a reference panel, and are provided by the Wellcome Trust Case Control Consortium (WTCCC). Two of these data sets contain genetic information from chromosome 22. Of these, the smaller dataset referred to as “Chr22” contains 500 markers, while the larger referred to as “Chr22_3841” contains 3841 markers. Both studies include the genetic information of 1093 sample genotypes. The latter set is the same set that is used in ref. 6 for the study of ImputeV2.

Tables 1 and 2 give allelic-imputation error rate of Chr22 and Chr22_3841 respectively for each haplotype-inference method. Those results suggest that both LRMC-s and MCCF algorithms consistently outperform Beagle for both data sets regardless whether or not the missing proportion is low or high. For the approaches based on the matrix co-clustering factorization, BCU-2 and BCU-3 usually achieve the least error rate.

The third set, which is referred to as “WTCCC” in the study, includes one thousand and five hundred samples from the British Birth Cohort with 10,000 alleles for each. These 10,000 markers were taken from the middle of the entire genome-wide association study.

The error rates of various imputation approaches are summarized in Table 3. Similar to the results of previous tests, the best-performing approaches are BCU-2 and BCU-3. The low-rank matrix completion method performs better than Beagle but slightly worse than the best two co-clustering factorization algorithms.

#### Comparison in the Presence of Reference Panel

In the previous tests, we did not provide any reference information when performing imputation, which however is not the case in many imputation studies. To further justify the capability of our approaches, we run our test on a dataset that includes reference information. This dataset is from the HapMap3, and itself consists of 165 sampled individuals with 19711 alleles per sample. For the purpose of imputation, 41 of the samples were separated to form a reference panel, and we had the remaining 124 samples gradually masked (from 5% to 90%) as the testing data.

The error rates of the imputation methods discussed in this paper are presented in Table 4.

It appears in this case that both LRMC-s and MCCF algorithms significantly outperforms other methods. In the presence of reference panels, Beagle is superior to all of our matrix co-clustering factorization based approaches. However, we notice that the dataset under test is not balanced in its two dimensions. In other words, we are dealing with a very unbalanced matrix with almost 20000 markers and only hundreds of samples.

Therefore, to further investigate the impact of reference information on our approaches, we keep the number of samples unchanged while selecting only the first 3000 markers, even further only the first 300 markers, and run similar tests under new settings respectively. The error rates of each imputation algorithm are reported in Tables 5 and 6. From those tables, we can see that the performances of our matrix co-clustering factorization based algorithms steadily improve as the number of markers decreases. When considering only 300 markers, all of our proposed algorithms enjoy a lower error rate than that of Beagle.

#### Comparison under some more practical scenarios

In this section, we generated a few new data sets to further compare the error rates given by Mendel-Impute, LRMC-s and MCCF, and these results are reported in Table 7; and the CPU time consumed is given in Table 8. Specifically, we generated 1% missing rate data for chr22, chr22_3841, WTCCC and HapMap3 respectively. In addition, we generated 1KG_chr22, which consists 60000 SNPs and 1092 individuals. We took the first half of individuals as reference panel and masked about 85% the second half individuals as study panel. Then we tested this data set either with reference or without reference panel. It is worth mentioning that the resulting matrices by masking components through these two ways are more likely to be encountered in practice.

The dataset of 1KG_chr22 setup information is detailed as follows. The dataset 1KG_chr22 was prepared to represent imputing SNPs from a reference panel typed on a different chip from the study sample, as described in ref. 4. We assumed that the data were generated by the Illumina “Infinium Omni2.5-8 BeadChip”. We created a study and reference panel derived from the 1000 Genomes Project haplotypes from the March 2012 release of Phase 1, obtained from the IMPUTE2 website^2^. This dataset consists of haplotypes for 1092 individuals, which we split into a study and reference panel by assigning half of the individuals to each such that the distribution of ethnicities was preserved across both groups. For our study panel we chose 60,000 SNPs from a randomly selected region on chromosome 22 and masked genotypes of all SNPs that were not present in Illumina’s manifest file for the Omni2.5-8 Beadchip. The resulting study panel included 8,808 SNPs for which genotypes were present, and 51,192 SNPs with masked genotypes. Thus, the study panel consists of 85.3% systematically missing data for 546 individuals.

For MCCF algorithms, we used *k*_1_ = 100 and *k*_2_ = 20. The error rates of different algorithms are summarized in Table 7. All the experiments in this subsection were done on a multicore computer with eight 2.8 GHz Intel Core E3 processors and 16 GB of RAM.

From Table 7 we see that LRMC-s and Mendel achieve comparable error rates, both outperforming Beagle and fastPHASE in all scenarios. Moreover, for the biggest data set 1KG_chr22 with reference panel (a matrix of size 60000 × 2184), the error rate of LRMC-s is slightly lower than that of Mendel. Since there are markers with all samples missing in the study panel of 1KG_chr22, both fastPHASE and Beagle failed to return a valid phased file when they are not provided with reference panel.

Since it is reported in ref. 4 that Mendel is very efficient in terms of running time (much faster than Beagle, especially when the data set is large), we also compared the CPU times of Mendel-Impute and our algorithms. The running times are recorded in seconds using the tic/toc functions of MATLAB.

Table 8 suggests that BCU-2 and BCU-3 are the fastest algorithms. Combining with Table 7, we note that their yielded error rates are not as good as that of Mendel-Impute and LRMC-s but the differences are relatively small. Moreover, their error rates are better than Beagle’s in most cases. In addition, Mendel is usually faster than LRMC-s. However, for the 1KG_chr22 data set without reference, LRMC-s is faster than Mendel.

For general memory usage, we tested our algorithms with the dataset 1KG_chr22 with reference panel (which is a matrix of 60000*2100 entries). It shows that the peak memory usage of our algorithms is about 1 GB, which matches the size of the input matrix representation with MATLAB.

### Robustness of our MCCF algorithms with different values of *k*_1_ and *k*_2_

Tables 9, 10, 11, 12, 13 show the error rates of the four MCCF algorithms with different combinations of *k*_1_ and *k*_2_ for datasets Chr22, Chr22_3841, WTCCC and HapMap3 with 1% missing entries, and 1KG_chr22.

From Tables 9, 10, 11, 12, 13 we can observe that in terms of error rate, MCCF is not sensitive to *k*_1_, while larger *k*_2_ usually produces smaller error rate (the only exception is MBI_BL for 1KG_chr22 where k2 = 2 gives better error rate than larger ones, but the difference is not very significant). Moreover, it is intuitive that larger *k*_2_ usually results in longer CPU time, because larger linear systems are needed to be solved in the MCCF algorithms. Therefore, to balance the tradeoff of error rate and running time, in practice we may choose BCU-2 and BCU-3 with *k*_1_ = 50 or 100, and *k*_2_ = 5.

## Summary and Discussion

In this study, we present an observation regarding the underlying structural property of the large, noisy GWAS genetic data matrices with missing data: these data matrices may contain the sparse information of low-rank and low number of co-clusters. Chi *et al*.^4^ have developed Mendel-Impute as a low-rank matrix completion model for GWAS imputation. For the next-generation genotype imputation, we believe that further mathematical or statistics model design and new method development should make use of this sparsity structure.

With this paper, we present SparRec: a new framework for genotype imputation based on our study of the sparse properties of genetic data matrices. The computational models of SparRec are based on low-rank and low number of co-clusters of GWAS data matrices, and the performance is better than or comparable to the current state-of-the-art methods for GWAS imputation. As with other matrix completion based methods, such as Mendel-Impute, SparRec does not require the information of reference panels, and it can naturally handle large and complex missing data imputation tasks, which may contain multiple reference panels and diverse study panels from different platforms with different sets of SNPs. While IMPUTEv2 tried to handle datasets with unphased and incomplete genotypes from different platforms (refer to scenario 1 and scenario 2 of IMPUTEv2^2^), its model may not be able to accommodate scenarios other than the two scenarios considered in ref. 2, and it is not convenient to prepare for the required input file formats for IMPUTEv2. Also note that current imputation methods, such as Beagle, can work without a reference panel; however it does not work with two or more haplotype or genotype reference panels used in the same run. Compared with the state-of-the-art approaches, SparRec is user-friendly and there is no limit on the number of references or study panels for the metadata analysis. To further enhance GWAS capabilities and facilitate downstream analysis, SparRec offers a viable alternative for biomedical researchers to perform various imputation tasks.

From a methodological point of view, current popular statistics imputation methods, such as IMPUTE v1^17^, IMPUTE v2.2^2^, MACH^18^, fastPHASE^6^ and Beagle^7^, are based on using “local” structure information of the reference panels, and the genotype imputation is modeled as a “mosaic” of patterns in relation to the haplotype reference panels. For this reason, current statistics methods usually handle only one study dataset at a time with only one or two reference panels^1^,^3^. In contrast, Mendel-Impute^4^ and our newly proposed LRMC-s method use information based on low-rank structure of the data. From that point of view, Mendel-Impute and our method could naturally utilize all available information of the reference panels and the study panels to impute the missing data entries. Moreover, our MCCF method takes advantage of the low co-cluster number property, which is also the structural information of the dataset. Interestingly, even with missing data rates as high as 90%, our methods could still reliably achieve high level of imputation accuracy because the data matrix does possess a clear sparse/low-rank structure.

## Additional Information

**How to cite this article**: Jiang, B. *et al*. SparRec: An effective matrix completion framework of missing data imputation for GWAS. *Sci. Rep.* **6**, 35534; doi: 10.1038/srep35534 (2016).

## Figures and Tables

**Table 1:** Error rate for estimation of missing genotypes for Chr22.

Method	Error Rate							
	5% Masked	10% Masked	25% Masked	50% Masked	60% Masked	70% Masked	80% Masked	90% Masked
fastPHASE	0.1152	0.1202	0.1216	0.1590	0.1665	0.1810	0.1766	0.1900
Beagle	0.1071	0.1464	0.1285	0.1797	0.1996	0.2016	0.2032	0.2066
Mendel	0.0106	0.0104	0.0146	0.0202	0.0263	0.0358	0.0507	0.1160
LRMC-s	0.0090	0.0110	0.0129	0.0200	0.0248	0.0329	0.0464	0.1350
BCU-1	0.0174	0.0170	0.0215	0.0308	0.0374	0.0510	0.0670	0.1315
BCU-2	0.0176	0.0166	0.0207	0.0309	0.0370	0.0522	0.0661	0.1274
BCU-3	0.0176	0.0166	0.0207	0.0309	0.0370	0.0522	0.0661	0.1274
MBI-BL	0.0174	0.0159	0.0191	0.0299	0.0349	0.0477	0.0701	0.1531

**Table 2:** Error rate for estimation of missing genotypes for Chr22_3841.

Method	Error Rate							
	5% Masked	10% Masked	25% Masked	50% Masked	60% Masked	70% Masked	80% Masked	90% Masked
fastPHASE	0.1189	0.1125	0.1739	0.1791	0.1821	0.1840	0.1793	0.1834
Beagle	0.0766	0.0735	0.0752	0.0819	0.0917	0.0950	0.0970	0.0951
Mendel	0.0148	0.0150	0.0163	0.0197	0.0218	0.0256	0.0330	0.0554
LRMC-s	0.0146	0.0148	0.0161	0.0194	0.0214	0.0251	0.0328	0.0578
BCU-1	0.0445	0.0446	0.0446	0.0451	0.0454	0.0462	0.0478	0.0569
BCU-2	0.0445	0.0446	0.0446	0.0451	0.0454	0.0462	0.0479	0.0567
BCU-3	0.0445	0.0446	0.0446	0.0451	0.0454	0.0462	0.0479	0.0567
MBI-BL	0.0444	0.0446	0.0447	0.0453	0.0457	0.0466	0.0488	0.0688

**Table 3:** Error rate for estimation of missing genotypes for WTCCC data.

Method	Error Rate							
	5% Masked	10% Masked	25% Masked	50% Masked	60% Masked	70% Masked	80% Masked	90% Masked
fastPHASE	0.2658	0.2865	0.3115	0.3130	0.3135	0.3154	0.3165	0.3157
Beagle	0.2383	0.2366	0.2312	0.2228	0.2193	0.2159	0.2126	0.2095
Mendel	0.1666	0.1665	0.1667	0.1676	0.1688	0.1715	0.1760	0.1888
LRMC-s	0.1667	0.1668	0.1681	0.1714	0.1736	0.1772	0.1825	0.1927
BCU-1	0.1648	0.1643	0.1645	0.1649	0.1648	0.1668	0.1651	0.1656
BCU-2	0.1648	0.1643	0.1645	0.1648	0.1648	0.1668	0.1651	0.1656
BCU-3	0.1648	0.1643	0.1645	0.1648	0.1648	0.1668	0.1651	0.1656
MBI-BL	0.1649	0.1644	0.1646	0.1649	0.1648	0.1685	0.1652	0.1659

**Table 4:** Error rate for estimation of missing genotypes for HapMap3 data with 19711 markers.

Method	Error Rate							
	5% Masked	10% Masked	25% Masked	50% Masked	60% Masked	70% Masked	80% Masked	90% Masked
fastPHASE	0.2090	0.1860	0.1730	0.3140	0.3120	0.3130	0.3140	0.3160
Beagle	0.1335	0.1369	0.1351	0.1469	0.1560	0.1649	0.1762	0.2000
Mendel	0.0251	0.0282	0.0349	0.0530	0.0638	0.0790	0.1022	0.1525
LRMC-s	0.0247	0.0277	0.0340	0.0518	0.0628	0.0780	0.1007	0.1481
BCU-1	0.1663	0.1682	0.1705	0.1729	0.1760	0.1805	0.1835	0.1850
BCU-2	0.1664	0.1681	0.1706	0.1729	0.1760	0.1806	0.1833	0.1850
BCU-3	0.1664	0.1681	0.1706	0.1729	0.1760	0.1806	0.1833	0.1850
MBI-BL	0.1663	0.1683	0.1710	0.1735	0.1769	0.1812	0.1835	0.1850

**Table 5:** Error rate for estimation of missing genotypes for HapMap3 data with 3000 markers.

Method	Error Rate							
	5% Masked	10% Masked	25% Masked	50% Masked	60% Masked	70% Masked	80% Masked	90% Masked
fastPHASE	0.2329	0.1978	0.3174	0.3085	0.3047	0.3070	0.3066	0.3055
Beagle	0.1323	0.1335	0.1353	0.1496	0.1596	0.1696	0.1761	0.1933
Mendel	0.0300	0.0330	0.0394	0.0572	0.0677	0.0820	0.1034	0.1510
LRMC-s	0.0296	0.0320	0.0384	0.0562	0.0670	0.0809	0.1018	0.1469
BCU-1	0.1377	0.1398	0.1428	0.1459	0.1504	0.1574	0.1684	0.1816
BCU-2	0.1377	0.1392	0.1429	0.1459	0.1502	0.1570	0.1690	0.1816
BCU-3	0.1377	0.1392	0.1429	0.1459	0.1502	0.1570	0.1690	0.1816
MBI-BL	0.1388	0.1400	0.1425	0.1463	0.1508	0.1572	0.1679	0.1815

**Table 6:** Error rate for estimation of missing genotypes for HapMap3 data with 300 markers.

Method	Error Rate							
	5% Masked	10% Masked	25% Masked	50% Masked	60% Masked	70% Masked	80% Masked	90% Masked
fastPHASE	0.2864	0.2241	0.2591	0.3206	0.3264	0.3225	0.3331	0.3565
Beagle	0.1454	0.1357	0.1488	0.1533	0.1666	0.1747	0.2027	0.2272
Mendel	0.0292	0.0372	0.0445	0.0683	0.0819	0.1016	0.1274	0.1803
LRMC-s	0.0280	0.0366	0.0441	0.0685	0.0826	0.1018	0.1275	0.1765
BCU-1	0.0847	0.0975	0.1014	0.1048	0.1163	0.1319	0.1481	0.1940
BCU-2	0.0847	0.0980	0.1008	0.1043	0.1150	0.1310	0.1484	0.1953
BCU-3	0.0847	0.0980	0.1008	0.1043	0.1150	0.1310	0.1484	0.1953
MBI-BL	0.0844	0.0963	0.0991	0.1041	0.1135	0.1268	0.1469	0.1928

**Table 7:** Error rates of our algorithms, Mendel-Impute, fastPhase and Beagle on various data sets.

	1% Masked			1% Masked HapMap3 With Ref.			1KG_chr22	
	chr22	chr22_3841	WTCCC	19711 M.	3000 M.	300 M.	With Ref.	No Ref.
fastPHASE	0.1288	0.0759	0.2507	0.2185	0.1465	0.1662	0.0668	———
Beagle	0.1266	0.0757	0.2386	0.1279	0.1229	0.1447	0.0359	———
Mendel	0.0090	0.0146	0.1665	0.0237	0.0278	0.0267	0.0252	0.0710
LRMC-s	0.0090	0.0145	0.1665	0.0223	0.0267	0.0279	0.0227	0.0710
BCU-1	0.0147	0.0455	0.1641	0.1639	0.1354	0.0812	0.0391	0.0710
BCU-2	0.0147	0.0456	0.1641	0.1639	0.1354	0.0812	0.0436	0.0710
BCU-3	0.0147	0.0456	0.1641	0.1639	0.1354	0.0812	0.0428	0.0710
MBI-BL	0.0147	0.0455	0.1641	0.1637	0.1377	0.0749	0.0680	0.0710

**Table 8:** CPU times (in seconds) of our algorithms and Mendel-Impute on various data sets.

	1% Masked			1% Masked HapMap3 With Ref.			1KG_chr22	
	chr22	chr22_3841	WTCCC	19711 M.	3000 M.	300 M.	With Ref.	No Ref.
Mendel	2.87	303.76	1198.24	170.85	39.23	4.70	9134.84	1419.79
LRMC-s	1.67	378.63	1333.61	299.69	51.83	2.67	15183.58	1143.91
BCU-1	1.66	116.59	506.59	126.31	30.10	2.89	4996.243	253.57
BCU-2	0.39	21.55	94.28	25.46	5.98	0.61	2381.207	139.84
BCU-3	0.41	23.30	101.70	27.51	6.48	0.70	2639.499	159.55
MBI-BL	2.84	286.24	1070.24	295.66	46.78	3.71	8259.953	395.87

**Table 9:** Error rate of MCCF with different *k*_1_ and *k*_2_ (dataset: Chr22, 1% masked).

*k*_2_	*k*_1_ = 50					*k*_1_ = 100					*k*_1_ = 200				
	2	3	5	10	20	2	3	5	10	20	2	3	5	10	20
BCU-1	0.11	0.08	0.05	0.02	0.01	0.11	0.08	0.05	0.02	0.01	0.11	0.08	0.05	0.02	0.01
BCU-2	0.11	0.08	0.05	0.03	0.01	0.11	0.08	0.05	0.03	0.01	0.11	0.08	0.05	0.03	0.01
BCU-3	0.11	0.08	0.05	0.03	0.01	0.11	0.08	0.05	0.03	0.01	0.11	0.08	0.05	0.03	0.01
MBI-BL	0.11	0.08	0.05	0.03	0.01	0.11	0.08	0.05	0.03	0.01	0.11	0.08	0.05	0.03	0.01

**Table 10:** Error rate of MCCF with different *k*_1_ and *k*_2_ (dataset: Chr22_3841, 1% masked).

*k*_2_	*k*_1_ = 50					*k*_1_ = 100					*k*_1_ = 200				
	2	3	5	10	20	2	3	5	10	20	2	3	5	10	20
BCU-1	0.08	0.08	0.07	0.06	0.05	0.08	0.08	0.07	0.06	0.05	0.08	0.08	0.07	0.06	0.05
BCU-2	0.08	0.08	0.07	0.06	0.05	0.08	0.08	0.07	0.06	0.05	0.08	0.08	0.07	0.06	0.05
BCU-3	0.08	0.08	0.07	0.06	0.05	0.08	0.08	0.07	0.06	0.05	0.08	0.08	0.07	0.06	0.05
MBI-BL	0.08	0.08	0.07	0.06	0.05	0.08	0.08	0.07	0.06	0.05	0.08	0.08	0.07	0.06	0.05

**Table 11:** Error rate of MCCF with different *k*_1_ and *k*_2_ (dataset: WTCCC, 1% masked).

*k*_2_	*k*_1_ = 50					*k*_1_ = 100					*k*_1_ = 200				
	2	3	5	10	20	2	3	5	10	20	2	3	5	10	20
BCU-1	0.16	0.16	0.16	0.16	0.16	0.16	0.16	0.16	0.16	0.16	0.16	0.16	0.16	0.16	0.16
BCU-2	0.16	0.16	0.16	0.16	0.16	0.16	0.16	0.16	0.16	0.16	0.16	0.16	0.16	0.16	0.16
BCU-3	0.16	0.16	0.16	0.16	0.16	0.16	0.16	0.16	0.16	0.16	0.16	0.16	0.16	0.16	0.16
MBI-BL	0.16	0.16	0.16	0.16	0.16	0.16	0.16	0.16	0.16	0.16	0.16	0.16	0.16	0.16	0.16

**Table 12:** Error rate of MCCF with different *k*_1_ and *k*_2_ (dataset: HapMap3, 1% masked).

*k*_2_	*k*_1_ = 50					*k*_1_ = 100					*k*_1_ = 200				
	2	3	5	10	20	2	3	5	10	20	2	3	5	10	20
BCU-1	0.18	0.18	0.18	0.17	0.16	0.18	0.18	0.18	0.17	0.16	0.18	0.18	0.18	0.17	0.16
BCU-2	0.18	0.18	0.18	0.17	0.16	0.18	0.18	0.18	0.17	0.16	0.18	0.18	0.18	0.17	0.16
BCU-3	0.18	0.18	0.18	0.17	0.16	0.18	0.18	0.18	0.17	0.16	0.18	0.18	0.18	0.17	0.16
MBI-BL	0.18	0.18	0.18	0.17	0.16	0.18	0.18	0.18	0.17	0.16	0.18	0.18	0.18	0.17	0.16

**Table 13:** Error rate of MCCF with different *k*_1_ and *k*_2_ (dataset: 1KG_Chr22).

*k*_2_	*k*_1_ = 50					*k*_1_ = 100					*k*_1_ = 200				
	2	3	5	10	20	2	3	5	10	20	2	3	5	10	20
BCU-1	0.05	0.05	0.04	0.04	0.04	0.05	0.05	0.04	0.04	0.04	0.05	0.05	0.04	0.04	0.04
BCU-2	0.05	0.05	0.05	0.05	0.05	0.05	0.05	0.04	0.05	0.05	0.05	0.05	0.04	0.05	0.05
BCU-3	0.05	0.05	0.05	0.04	0.04	0.05	0.05	0.04	0.04	0.05	0.05	0.05	0.04	0.05	0.05
MBI-BL	0.05	0.05	0.07	0.07	0.07	0.05	0.07	0.07	0.07	0.07	0.05	0.07	0.07	0.07	0.07
