# Corrigendum: SparRec: An effective matrix completion framework of missing data imputation for GWAS

**DOI:** 10.1038/srep37365

**Published:** 2016-11-21

**Authors:** Bo Jiang, Shiqian Ma, Jason Causey, Linbo Qiao, Matthew Price Hardin, Ian Bitts, Daniel Johnson, Shuzhong Zhang, Xiuzhen Huang

Scientific Reports 6: Article number: 35534; 10.1038/srep35534 published online: 10 20 2016; updated: 11 21 2016.

In the original version of this Article, Xiuzhen Huang was incorrectly affiliated to “College of Computer, National University of Defense Technology, Changsha 410073, China”. The correct affiliations are listed below:

Department of Computer Science, Arkansas State University, Jonesboro, Arkansas 72467, United States of America.

The UALR/UAMS Joint Graduate Program in Bioinformatics, Little Rock, Arkansas 72204, United States of America.

Molecular Biosciences Program, Arkansas State University, Jonesboro, Arkansas 72467, United States of America.

This error has now been corrected in the PDF and HTML versions of the Article.

